# Physician organization care management capabilities associated with effective inpatient utilization management: a fuzzy set qualitative comparative analysis

**DOI:** 10.1186/s12913-014-0582-5

**Published:** 2014-12-03

**Authors:** Thomas J Sheehy, N Marcus Thygeson

**Affiliations:** Children First for Oregon, Portland, Oregon, USA (formerly Goldman School of Public Policy, University of California, Berkeley, California USA; Healthcare Services Department, Blue Shield of California, San Francisco, California USA

## Abstract

**Background:**

We studied the relationship between physician organization (PO) care management capabilities and inpatient utilization in order to identify PO characteristics or capabilities associated with low inpatient bed-days per thousand.

**Methods:**

We used fuzzy-set qualitative comparative analysis (fsQCA) to conduct an exploratory comparative case series study. Data about PO capabilities were collected using structured interviews with medical directors at fourteen California POs that are delegated to provide inpatient utilization management (UM) for HMO members of a California health plan. Health plan acute hospital claims from 2011 were extracted from a reporting data warehouse and used to calculate inpatient utilization statistics. Supplementary analyses were conducted using Fisher’s Exact Test and Student’s T-test.

**Results:**

POs with low inpatient bed-days per thousand minimized length of stay and surgical admissions by actively engaging in concurrent review, discharge planning, and surgical prior authorization, and by contracting directly with hospitalists to provide UM-related services. Disease and case management were associated with lower medical admissions and readmissions, respectively, but not lower bed-days per thousand.

**Conclusions:**

Care management methods focused on managing length of stay and elective surgical admissions are associated with low bed-days per thousand in high-risk California POs delegated for inpatient UM. Reducing medical admissions alone is insufficient to achieve low bed-days per thousand. California POs with high bed-days per thousand are not applying care management best practices.

**Electronic supplementary material:**

The online version of this article (doi:10.1186/s12913-014-0582-5) contains supplementary material, which is available to authorized users.

## Background

Reducing or eliminating unnecessary inpatient care is highly desirable, for both financial and quality of care reasons. Hospital costs are high [1], rising rapidly [2], and widely viewed as unsustainable [3]. Inpatient facilities are also dangerous places for patients. The rates of “never events”, hospital acquired complications and infections, and other facility-related adverse health outcomes remain unacceptably high, despite a concerted effort in the last decade to reduce the occurrence of such events [4]. Thus, reducing avoidable inpatient bed-days remains a major focus of managed care companies, health plans, and government programs like Medicare.

Efforts to control health care costs have led to significant shifts in the make-up of the healthcare market since 1980, including the wide-spread adoption of a variety of managed care practices including utilization management, concurrent review, case management, discharge planning, and disease management. In addition, hospitalist physicians have assumed responsibility for a growing proportion of inpatient care [5]. Rather than building vertically integrated systems, non-Kaiser health management organization (HMO) plans in California have contracted with independent practice associations (IPAs) and medical groups, paying them capitation to cover the cost of a broad variety of outpatient services (but generally not inpatient services), and delegating them to actively manage inpatient care on the Plan’s behalf [6].

The continuing effectiveness of common care management strategies, particularly as it relates to hospital utilization and costs, is controversial. Preadmission and concurrent review of inpatient stays were initially viewed as effective, but evidence for continued cost-effectiveness has been questioned [7,8]. Discharge planning and transition of care management appear to reduce length of stay and readmission rates [9]. A recent Agency for Healthcare Research and Quality review of case management concluded that it may reduce re-hospitalization rates for patients at high risk for readmission, but has little effect on inpatient utilization for patients with chronic medical illness in general [10]. The impact of disease management programs on hospital admission rates is similarly controversial [7]. Hospitalist programs have been associated with shorter hospital length of stay but not reduced readmissions, mortality, or overall costs [11–14].

Most research in this area has been focused on evaluating the impact of specific care and utilization management programs. We were unable to find any published studies regarding which combinations of physician organization (PO) characteristics and medical management capabilities are currently associated with effective inpatient bed-day management. Prior work has indicated that care management by POs may shorten hospital length of stay, but little is known about the methods by which these results are achieved [15]. This study was designed to address this gap, and is analogous to a population-based study that evaluates the efficacy of interventions in actual practice settings, thereby providing useful information for policy and decision makers. This information is particularly relevant now because of the recent interest in accountable care organizations (ACOs) as an approach to managing health care cost and quality.

We adopted a set-theoretic approach, fuzzy set qualitative comparative analysis (fsQCA), for this study. fsQCA is especially well-suited for case-oriented, exploratory studies of social phenomena that manifest complex causality (characterized by configurational equifinality and causal asymmetry), where the number of cases is small (typically 10-50), the data and concepts are qualitative, and theory is not well developed [16–18]. fsQCA identifies associations (subset relationships) between independent conditions and dependent conditions (outcomes) in small case series that would often be missed using conventional statistical analysis. Also, fsQCA provides a method for calibration and quantification of inherently “fuzzy” qualitative data (fuzzy set membership assignment) that enables systematic analysis using both qualitative comparative analysis and standard statistical methods.

The central purpose of this study is to investigate which PO medical management practices are linked to effective inpatient utilization management, as manifested by low inpatient bed-days per thousand members per year (bed-days).

## Methods

### Data and study group

#### Case selection

POs invited to participate in the study are located in California, capitated for outpatient services, and delegated to provide inpatient utilization management. The health plan pays all facility claims directly. The invited POs met three additional criteria: above average population risk (average DxCG risk score—DxCG version 3.03—greater than or equal to the Plan average); only one geographic service area; and average annual Plan membership greater than or equal to 1,000. These requirements ensured that there would be sufficient variability in the dependent characteristic (bed-days) and reduced variability related to geographic practice pattern variation and small membership numbers, respectively. We had previously found that average bed-days and between-group variance in bed-days are both low when population risk is low (data not shown). Sixteen POs met all three of these criteria; fourteen (87.5%) responded to interview requests.

#### PO capabilities assessment

Medical directors for the participating POs were contacted to schedule an 84-question telephonic survey. (A copy of the survey is available in the supplemental online materials (Additional file 1)). Interviews were conducted with medical directors and relevant PO medical management staff. Each interview was conducted by the same investigator (Sheehy), in order to ensure consistent delivery of the instrument. The survey was exploratory in nature and focused on in- and out-patient management practices, including details of contracts and scope of practice agreements with hospitals and hospitalists; frequency and quality of pre-admission and concurrent review and discharge planning conducted by RNs, Medical Directors, and hospitalists; and case and disease management practices. Additional questions were included about internal management structures, including primary care-to-specialist ratio, governance structures, utilization of electronic health records and health information exchanges, and compensation systems including use of capitation, fee-for-service, and performance-based bonuses. Response rates differed widely on more financially or politically sensitive topics, especially those dealing with compensation. Consequently, this analysis focuses exclusively on utilization management practices, topics for which there are no missing data points.

#### Claims data

Inpatient claims for commercial HMO plan members assigned to the participating POs with dates of service in calendar year 2011 were used to calculate the dependent variables admissions per thousand (admits), bed-days per thousand (bed-days), and average length of stay (ALOS). We focused on bed-days the POs are expected to actively manage, and therefore only medical and surgical discharges from California acute-care facilities for diagnosis related groups (DRGs) included in the Milliman 2011 managed care inpatient utilization benchmark (2011 Milliman Commercial Health Cost Guidelines) were included in these calculations. Claims for maternity, newborn, and behavioural health discharges were excluded from the analysis, as were claims from out-of-state, skilled nursing, and long term acute care facilities. We also excluded claims for which the Plan was not the primary payer.

### Fuzzy set qualitative comparative analysis (fsQCA)

fsQCA is based on fuzzy set theory and logic, not statistical inference or correlations, and therefore is a fundamentally different approach from conventional linear, net-effects, regression analysis. Detailed reviews of the fsQCA method are available in several recent publications [17,19]. Here we briefly define key concepts to facilitate the comprehension of readers who are not familiar with the method, and describe specific aspects of our approach.

In conventional “crisp” set theory, set membership is binary—each case is either fully in or out of the set of cases with a given characteristic. For example, PO membership in the set of “POs with effective inpatient utilization management” may equal 1 or 0, respectively. In contrast, with fuzzy set theory, set membership may have intermediate values between 0 and 1. For instance, POs may be considered fully out (set membership equal 0), more out than in (set membership equal 0.33), more in than out (set membership equal 0.67), or fully in (set membership equal 1) the set of “POs with effective inpatient utilization management”.

Combinations of conditions (configurations) may be associated with outcomes. Set membership in individual component conditions can be combined in standard ways (e.g., intersection [OR] and union [AND]) to calculate set membership in the configuration. Multiple conditions can be combined and analyzed in this way. This characteristic of fsQCA is similar to interaction effects in regression analysis; but fsQCA has several advantages in this respect. First, fsQCA enables evaluation of interaction effects with small-N data sets. Second, fsQCA can be used to study interactions between three or more conditions, which is very difficult using regression. Truth table analysis is the method used in fsQCA to evaluate the relationship between multiple conditions and an outcome.

fsQCA identifies two types of set relationship—necessity and sufficiency. In a necessary relationship, cases with the outcome of interest are a subset of the cases with the condition(s) of interest. Cases with the outcome manifest the condition, but not all cases with the condition manifest the outcome. With a sufficient relationship, cases with the condition or characteristic of interest are a subset of the cases with the outcome of interest. The presence of the condition is sufficient to cause the achievement of the outcome. More than one sufficiency relationship is possible; there may be multiple paths to the same outcome (equifinality).

The strength and importance of the relationship between condition(s) and outcome is measured by two metrics, consistency and coverage, each of which may take values between 0 and 1. Consistency measures the degree to which the necessary or sufficient relationship holds true (one set is a subset of the other) and is comparable to a correlation coefficient. (Correlations are “both necessary and sufficient” relationships.) Coverage represents the degree to which a causal configuration accounts for (or “covers” if one thinks of a Venn diagram) the outcome, and is analogous to relevance. Causal configurations with high consistency but low coverage are analogous to interventions that are statistically significant but clinically irrelevant. Consistency of 0.8 is the generally accepted threshold for a potentially meaningful subset relationship between outcomes and conditions or configurations of conditions [19]. Unique coverage is the frequency with which a specific sufficient component of a multi-component causal or explanatory “recipe” is the only observed condition for the outcome [19].

Survey responses were calibrated according to the criteria listed in Additional file 2. We did not have *a priori* meaningful crossover thresholds for quantitative utilization data, and thus it was calibrated indirectly (Additional file 2).

We used STATA’s centroid linkage cluster-analysis function (“cluster” package) to sort uncalibrated values into six “buckets”, and each bucket was assigned a score between 0 and 1 in increments of 0.2. We then used the STATA “fuzzy” package’s indirect calibration function to assign a continuous fuzzy score based on those group scores (indirect calibration is discussed in greater detail by Ragin [19]). We combined related concepts using the fuzzy set operations union or intersection to prevent the number of explanatory conditions included in the analysis exceeding the limits suggested by Marx and Dusa [20]. The data and data dictionary used for this study are available as supplemental online materials (Additional file 3 and Additional file 4, respectively).

Prior to data collection we developed a logic model, shown in Additional file 5, outlining which PO practices (e.g. concurrent review) may affect the different components (e.g. medical ALOS) of the major outcome of interest, total bed-days. Total bed-days is the sum of medical bed-days and surgical bed-days. Medical and surgical bed-days are the product of medical and surgical admits and average length of stay, respectively.

We then conducted a sequential set of analyses. We first identified the set relationships between total bed-days and its separate components (intermediate outcomes) medical and surgical admits and ALOS. We then evaluated the set relationships between each intermediate outcome and the PO practices or characteristics designed to address each intermediate outcome. For instance, disease management is designed to reduce medical admits, whereas pre-service review (prior authorization) is primarily focused on reducing unnecessary surgical admits. Concurrent review and discharge planning are designed to reduce average surgical and especially medical ALOS (Additional file 5).

All fsQCA procedures were completed using the fs/QCA software [21]. In all truth table analyses the consistency threshold for reduction of solutions was set at 0.80, unless otherwise noted; the frequency threshold was one; and we report intermediate solutions that incorporate “easy” counterfactual assumptions, a standard part of the fsQCA method. Fisher’s exact tests and t-tests were used to evaluate dichotomized results to confirm fsQCA findings. For these tests, group scores were dichotomized by setting the outcome or solution score equal to one if the fuzzy membership was greater than 0.5 and zero otherwise.

This study did not involve research on human subjects and was performed in compliance with the Helsinki declaration. Only aggregated health plan operational and provider organization survey data were used in the analysis. Provider group medical directors and staff gave informed consent and participated voluntarily on condition of confidentiality. Provider group results were de-identified; and only de-identified results were shared with the participants.

## Results

Of the fourteen participating POs, ten were IPAs, two were IPAs with small integrated medical groups, and two were integrated groups with wrap-around IPAs. The average number of Plan members enrolled in the PO in 2011 ranged from 995 to 25,583. Ten were located in Northern California and 4 were in the southern part of the state. Managed bed-days ranged from 124.36 to 199.42. Additional file 6 includes descriptive statistics of bed-days, medical and surgical admits, and medical and surgical ALOS.

The first step in the analysis was to examine the bivariate relationships between bed-days and the intermediate outcomes of medical admits, medical ALOS, surgical admits, and surgical ALOS to identify which intermediate outcomes are most strongly associated with low bed-days (Table 1).

**Table 1:** **Set relationships between intermediate outcomes and bed-days per thousand**

	**Consistency**	**Coverage**
Medical length of stay	0.901	0.665
Surgical admissions	0.843	0.622
Surgical length of stay	0.813	0.677
Surgical readmissions	0.725	0.707
Medical admissions	0.718	0.718
Medical readmissions	0.685	0.620

Only three intermediate outcomes fell above the consistency threshold of 0.80: medical ALOS, surgical admits, and surgical ALOS. Management of medical admits was not consistently linked to low bed-days.

We used fuzzy set truth table analysis to determine the combinations of intermediate outcomes and PO characteristics that contribute to well-managed bed-days. Table 2 presents fsQCA solutions for six models: one for bed-days, one for each for the intermediate outcomes, and one for medical readmissions.

**Table 2:** **Results of fsQCA truth table analysis**

**Outcome**	**Conditions**	**Solution**	**Consistency**	**Raw coverage**	**Unique coverage**
Bed days per thousand	Medical Length of Stay	Well-Managed Medical Length of Stay AND Well-Managed Surgical Admissions, OR	0.935	0.405	0.139
	Surgical Length of Stay	Well-Managed Medical Length of Stay AND Well-Managed Surgical Length of Stay, OR	0.950	0.489	0.223
	Medical Admissions	Well-Managed Surgical Admissions AND Well-Managed Surgical Length of Stay AND Well-Managed Medical Admissions	0.988	0.343	0.092
	Surgical Admissions	Total Recipe:	0.926	0.720	
Medical length of stay	Number of hospitals in-area	Low number of hospitals AND Robust concurrent review procedure AND Strong hospitalist relationship AND Active PO discharge role, OR	0.832	0.377	0.227
	Concurrent review procedure	High number of hospitals AND Non-robust concurrent review procedure AND Strong hospitalist relationship AND Active PO discharge role	0.920	0.306	0.156
	Strength of hospitalist relationship	Total Recipe:	0.839	0.533	
	PO role in discharge planning				
Surgical admissions	Surgical readmission rate	Robust prior authorization procedure AND Low surgical readmission rate AND Lack of discharge notification to PCPs, OR	0.855	0.509	0.263
	Prior authorization procedure	Robust prior authorization procedure AND Discharge notification to PCPs AND Direct PO staff role in discharge planning process	0.836	0.448	0.202
	Discharge notification procedure	Total Recipe:	0.847	0.711	
	PO staff role in discharge planning process				
Surgical length of stay	Surgical readmission rate	Night/ED hospitalist coverage AND Active PO discharge role AND Low number of hospitals, OR	0.960	0.421	0.099
	Number of in-area hospitals	Night/ED hospitalist coverage AND Active PO discharge role AND Low surgical readmission rate	0.885	0.533	0.211
	Night/ED hospitalist coverage	Total Recipe:	0.901	0.632	
	PO role in discharge planning				
Medical admissions*	Medical readmission rate	High medical readmission rate AND High number of UCC hours AND Robust disease management program, OR	0.977	0.327	0.172
	Average number of urgent care center (UCC) open hours	Low medical readmission rate AND Non-robust disease management program, OR	0.918	0.439	0.284
	Disease management program	Total Recipe:	0.929	0.611	
	PO FTEs dedicated to case management				
Medical re-admissions	Average number of non-peak urgent care center (UCC) open hours	High number of non-peak UCC hours AND Strong hospitalist relationship AND Low case management FTEs, OR	0.920	0.288	0.136
	PO FTEs dedicated to case management	High case management FTEs AND Weak hospitalist relationship, OR	0.948	0.379	0.110
	Strength of hospitalist relationship	High case management FTEs AND Frequent MD rounding	0.895	0.420	0.188
	PO medical director rounds	Total Recipe:	0.904	0.717	

*The consistency cut-off threshold for the medical admission truth table analysis was set to 0.9; lowering the threshold to 0.8 yields a less parsimonious solution with lower consistency (0.913) and slightly higher coverage (0.640).

### Total bed-days analysis

Analysis of the outcome “low total bed-days” (bed-days as the outcome and medical and surgical ALOS and admits as the conditions) identified three possible “paths” to low bed-days, all with consistency greater than 0.9. POs with low bed-days manage medical ALOS and either surgical ALOS or admits, or surgical and medical admits in combination with surgical ALOS. As indicated by the unique coverage scores, the most common paths to controlling bed-days are managing medical ALOS and either surgical ALOS or surgical admits. The coverage for these combined configurations is 0.628. In contrast, tight management of surgical ALOS and both medical and surgical admits has a unique coverage of 0.092 and is less empirically important. The combination of well-managed surgical and medical ALOS is the configuration with the highest raw and unique coverage, indicating that managing ALOS, rather than minimizing admissions, is the most common path to successful inpatient management. Highlighting the importance of ALOS, POs with high bed-days had low medical admissions but high ALOS and surgical admits (data not shown).

### Medical length of stay analysis

A second model evaluated the relationship between medical ALOS as the outcome and the number of in-area hospitals, concurrent review procedure, strength of PO-hospitalist relationship, and PO role in discharge planning as the conditions. The most important identified factors in controlling medical ALOS were a strong PO-hospitalist relationship and a robust role in discharge planning by PO staff or surrogates. Regardless of the number of hospitals, successful POs had both a contract and Scope of Practice agreement with a group of hospitalists and regularly reviewed data related to utilization management of their patients managed by the hospitalists. Additionally, all POs with low medical ALOS had staff actively participating in discharge planning and a Scope of Practice agreement with hospitals that detailed the discharge planning processes and roles and responsibilities of PO and hospital staff. Low ALOS POs with a small number of hospitals had directly-employed staff doing concurrent review on-site at each hospital five days a week. We found only one PO with a large number of hospitals successfully managing medical ALOS. In this case, only some concurrent review was on-site. However, the less intense off-site concurrent review was done seven days a week and there was full PO control over the patient discharge process.

### Surgical admissions analysis

The third model identified solution paths sufficient for achieving the outcome of well-managed surgical admits, given the conditions of the surgical readmission rate, prior authorization and discharge notification procedures, and PO staff role in the discharge process. An effective prior authorization process is a component in both of the sufficient configurations identified in this model and therefore is necessary for managing surgical admits. In addition to effective prior authorization, the staff of POs with low surgical admits are actively involved in the discharge planning process and regularly notify patients’ primary care physicians of discharge or, in the absence of an active PO role in discharge planning, have well-managed surgical readmissions.

### Surgical length of stay analysis

Evaluation of the association between well-managed surgical ALOS (outcome) and surgical readmission rate, number of in-area hospitals, hospitalist coverage at night or in the Emergency Department (ED), and the PO role in discharge planning (conditions) identified that hospitalist coverage at night or in the ED and an active PO role in discharge are both necessary conditions for well-managed surgical ALOS. The combination of these conditions with either a small number of in-network hospitals or a low rate of surgical readmissions is sufficient for low surgical length of stay.

### Medical admissions analysis

Although low medical admits was not consistently associated with low bed-days, we nevertheless evaluated which PO characteristics and practices are associated with low medical admission rates. We found two sufficient paths to well-managed medical admits: either low medical readmissions and non-robust disease management, or high medical readmissions combined with a robust disease management and high urgent care availability. There appear to be two dominant paths to low medical admits in this group of POs—either having low rates of medical readmissions, or using strong disease management and urgent care availability to reduce primary medical admissions. Having a high number of case managers was not linked directly to low medical admissions, although it was associated with low medical readmissions (see below).

Despite the association with low medical admissions, strong disease management programs were not invariably associated with low inpatient bed-days. Nine POs had disease management programs (fuzzy membership >0.5), and six of them had evidence of possible program effectiveness (medical admits fuzzy membership >0.5). Of these six, only two had low bed-days. The two POs with disease management programs and good performance on both admits and bed-days had evidence of strong engagement in at least two of the three activities related to ALOS management (concurrent review, discharge planning, and hospitalist contracting). The other four POs with disease management programs and low medical admits had high bed-days and fuzzy membership less than 0.5 in at least two of the three activities related to ALOS management.

### Medical readmission analysis

The final model evaluated the association between the outcome of medical readmission rates and the conditions of case management staffing ratios, hospitalist program effectiveness, urgent care availability after hours, and the frequency of PO medical director hospital rounds. All four capabilities appear to contribute in various combinations to lower medical readmissions. Communities with readily available urgent care after hours and effective hospitalist programs have low medical readmission rates despite low case management staffing. Where hospitalist resources are weak, high case management staffing is associated with low medical readmission rates. Finally, some POs achieved low medical readmission rates by combining medical director rounding and high case management staffing. High case management staffing was a component of the two configurations with the highest coverage, with a total coverage of 0.581, suggesting that it is an important contributor to low medical readmissions.

### Statistical analyses

Standard statistical methods confirmed the fsQCA results. As shown in Table 3, bed-days were significantly lower in “well-managed” POs with set membership scores greater than 0.5 in any one of the configurations (solutions) identified in the “total bed-days” model, and in POs with membership greater than 0.5 in the set “low surgical length of stay.” (See the Additional file 1 for details of variable coding and analysis). There was also a trend towards lower bed-days in groups with low medical length of stay. Average bed-days were arithmetically lower in groups with low surgical admits, but arithmetically higher in groups with low medical admits.

**Table 3:** **Difference in means, well-managed versus not well-managed**

**Physician organization set**	**Mean bed-days**	**Mean bed-days**	**Difference (Standard error)**
	**Well-managed**	**Not well-managed**	
Well-managed (any solution path)	138.47	165.62	-27.16
	(n =6)	(n =8)	(10.02)***
Low medical length of stay	141.29	161.04	-19.75
	(n =5)	(n =9)	(11.84)*
Low surgical admits	143.97	159.55	-15.58
	(n =5)	(n =9)	(12.34)
Low surgical length of stay	143.67	164.30	-20.63
	(n =7)	(n =7)	(11.09)**
Low medical admits	157.24	142.06	15.17
	(n =11)	(n =3)	(14.70)

***= p <0.01; **= p <0.05; *= p <0.1 (one-tailed).

Furthermore, after dichotomizing membership in the set “low bed days per thousand,” we found that being well-managed (high score on any of the solution paths identified in the “low total bed-days” model) or having low medical length of stay was sufficient for, and significantly associated with, low bed-days (p = 0.009 and 0.028, respectively, by Fisher exact test; see Online Appendix Additional file 6 for details).

Including illness burden (DxCG) in the medical and surgical admits models decreased solution consistency scores and did not materially change our findings (data not shown). Of the six POs with very high risk populations (fuzzy DxCG membership >0.5), five had well-managed medical admissions (fuzzy membership >0.5). There was no significant difference in the average bed-days fuzzy set membership between slightly high risk and very high risk POs (0.631 and 0.559, respectively; one-tailed t-test p = 0.3446), nor were bed-days significantly correlated with DxCG score (r = 0.314; one-tailed t-test p = 0.14).

## Discussion

Using fsQCA we identified a set of medical management practices that California physician organizations use in varying combinations to effectively manage hospital utilization. Groups with low bed-days generally use multiple strategies and are especially effective at managing medical ALOS. Specific PO practices that are closely linked to low medical ALOS include directly contracting and maintaining scope of practice agreements with local hospitalists and hospitals, and active PO involvement in both concurrent review and discharge planning. Effective concurrent review generally involved daily on-site review by nursing staff employed by the PO. These results are consistent with our theoretical expectations that rigorous PO management of the inpatient stay is associated with shorter length of stay.

We also found that POs with low surgical admits have effective prior authorization programs and are actively involved in discharge planning. Active PO involvement in discharge planning is also associated with low surgical length of stay. POs with low surgical length of stay also have hospitalist coverage at night or in the emergency department (ED). The mechanism of the latter association is not immediately evident, but if hospitalist night and ED coverage is linked to broader hospitalist scope of practice at a given hospital, this association may be related to and mediated by greater hospitalist participation in the post-operative care of surgical patients.

Strong case management programs, as manifested by high staffing ratios, and robust hospitalist programs were associated with low medical readmissions; and strong disease management programs were associated with low medical admissions. However, neither case nor disease management was associated with low total bed-days in the absence of effective management of length of stay.

Summarizing the above results, we found that close PO oversight of patient care from admission to discharge, strong contractual relationships with local hospitalists and hospitals, and effective pre-surgical prior authorization programs are the care management strategies most strongly associated with successful hospital utilization management in delegated POs. Case and disease management are effective at reducing medical readmissions and admissions, respectively. These findings indicate that many standard medical management practices remain effective when properly implemented by POs. Our results confirm earlier studies documenting the effectiveness of these approaches for reducing hospital utilization, and suggest that poor control of inpatient bed-days by POs is linked to failure to implement well-established medical management best practices.

The relationship between medical admits and bed-days was surprisingly weak, given the conventional wisdom that decreasing medical admissions through greater care coordination and disease management is key to lowering healthcare costs [22,23]. While PO disease management programs were associated with lower overall medical admits, POs with apparently effective disease management programs do not achieve low bed-days unless they also have effective programs for managing ALOS. The medical director of one of the POs told us they do not “micro-manage” inpatient care, choosing to instead focus on demand management strategies. This statement illustrates a trade-off between managing medical admits and ALOS. Some of this trade-off appears to be ideological, as suggested by the quotation above, but it may also be related to resource constraints. Concurrent management of both admits and ALOS should have a multiplicative effect on lowering bed-days, as indicated in the logic model used for this analysis (Additional file 5). However, managing medical admits but not medical ALOS was not a path to controlling bed-days in this group of POs.

Higher than average population risk was a criterion for PO inclusion in this study because we had observed that POs with low risk populations had low bed-days (i.e., low illness burden is a sufficient cause for low bed-days). Among this group of POs with above average illness burden, POs with both very high and only slightly high DxCG scores achieved similar bed-days with effective care management programs. Adding DxCG score to the models in Table 2 did not produce materially different solutions, nor was DxCG score significantly correlated with bed-days. Based on these findings, “my patients are sicker” does not appear to be a valid explanation for the variation in inpatient utilization observed among these POs with above average population risk. High illness burden is a necessary cause of high inpatient utilization; but it is not sufficient, and can be largely compensated for with good inpatient utilization management. There appear to be two sufficient “paths” to low bed-days—low population illness burden, or high illness burden and effective inpatient UM.

Our decision to use fsQCA for this study was validated in that we identified a variety of equifinal sufficient configurations of PO care management capabilities associated with low inpatient utilization. This study adds to the growing literature documenting the utility of fsQCA for program evaluation and health services research [24].

Limitations of this study include its narrow scope. We only included California POs delegated for inpatient utilization management of commercial HMO patients; we did not evaluate the cost-efficiency of the care management practices or the impact on health care costs; and we did not include contextual factors like PO contracting strategies, board composition, provider mix, or population demographics in our analysis.

We may not have identified all combinations of characteristics associated with low bed-days, ALOS, and admits. The coverage of the explanatory “recipes” we identified varied from 0.533 to 0.717, indicating that other strategies or characteristics may enable some POs to achieve low bed-days.

The structured interview instrument used to collect information about PO characteristics and capabilities has not been subjected to rigorous validity testing, which may have introduced unintended “noise” into our data. In such a small-N analysis, differences in how words or questions were understood by respondents (e.g. “scope of practice”) could be particularly impactful. However, our survey process was conducted by one person and was interactive, factors designed to minimize unwarranted variability in responses.

The health plan also provides case and disease management services to PO patients. Our study ignores any possible effect from these programs. Our findings represent the incremental impact of PO case and disease management programs, in addition to whatever impact the health plan programs had.

Like all analytic methods, fsQCA has certain limitations. There are no *ceteris paribus* assumptions or ways to control for endogeneity. The fsQCA analytic process is iterative, and requires that the investigator make certain decisions, such as selection of consistency thresholds and prime implicants, that are based on judgment and can introduce bias into the analysis. However, we planned, conducted, and documented the analytic process carefully, and followed standard fsQCA conventions, so as to minimize this risk. Our findings are further validated by supplemental statistical analyses showing that many of the configurations of medical management capabilities identified using fsQCA were significantly associated with low bed-days. Consequently, while our findings may be viewed as preliminary, we believe they are valid.

Our results suggest at least three fruitful areas for further research. First, in the analysis of the intermediate outcomes we found a highly sufficient relationship between well-managed bed-days as an outcome and well-managed ED visits per thousand members as a condition (consistency = 0.87). We believe that this relationship warrants further research to understand how low rates of ED use may be sufficiently linked to low bed-days. The association does not appear to be mediated by admits, because we found no association between ED visits and either medical or surgical admits. We speculate that low ED visits and low bed-days may both be the consequence of access to high-quality primary care services, but this must be tested empirically.

Second, we did not identify all configurations of conditions associated with successful inpatient utilization management; further work needs to be done here. Finally, although we identified several PO programs that are associated with low inpatient utilization, we have the opportunity to look at more detail at the characteristics of these programs in high-performing POs so as to better understand which specific behaviours and processes are particularly important. Further research into such “best-practices” would enable POs and hospitals to focus their efforts and resources on implementing the most effective programs. With that in mind we are using these results to refine our own inpatient medical management programs and ensure that POs delegated by us for inpatient utilization management have implemented the programs and practices we found most effective in this study.

Our findings have policy implications as well. As the healthcare system in the United States undergoes rapid change in response to the Affordable Care Act, policy makers should keep in mind that utilization management methods such as concurrent review, discharge planning, strong hospitalist programs, and prior authorization for elective surgical admissions play important roles in reducing potentially harmful avoidable inpatient bed-days and are necessary components of high quality managed care programs.

## Conclusions

Active participation in concurrent review and discharge planning for all admissions, strong hospitalist contracts and scope of practice agreements, and robust prior authorization for surgical admissions were the most effective care management strategies used by delegated California POs to reduce avoidable inpatient days and successfully manage hospital utilization. Disease and case management were associated with reduced medical admissions and readmissions, respectively, but were not sufficient for achieving low inpatient utilization. Managing length of stay is essential to achieve low bed-days per thousand. Incomplete implementation of medical management best practices by delegated physician organizations with high-risk populations is an important contributor to avoidable hospital utilization in a commercial California HMO plan.

## Additional files

Additional file 1:
**Structured interview questions_Sheehy_Thygeson.**
Additional file 2:
**Table S1_Sheehy_Thygeson.**
Additional file 3:
**Data_Sheehy_Thygeson.**
Additional file 4:
**Data dictionary_Sheehy_Thygeson.**
Additional file 5:
**Figure S1_Sheehy_Thygeson.**
Additional file 6:
**Online Appendix_Sheehy_Thygeson.**
