# Supplementary material for: Physician organization care management capabilities associated with effective inpatient utilization management: a fuzzy set qualitative comparative analysis
Source: BMC Health Serv Res. 2014 Dec 3;14:582. doi: 10.1186/s12913-014-0582-5 (PMC4263202; doi:10.1186/s12913-014-0582-5)
Supplement: Additional file 1: — Structured interview questions_Sheehy_Thygeson. [file 12913_2014_582_MOESM1_ESM.docx]

**1. What is your full name?**

**2. What is the job title for your current position?**

**3. What is the name of the IPA/MG for which you are employed?**

**4. Which of the following best describes your organization?**

mlj

IPA

mlj

Medical Group

mlj

Medical Group with a wraparound IPA

mlj

Other (please specify)

**5. Please explain the wraparound IPA structure (e.g. only specialists are part of the IPA,**

**specialists and some PCPs are part of the IPA, etc)**

5

6

**6. How many physicians were directly employed by the Medical Group in 2011?**

**7. What type of prior authorization criteria did you use in 2011?**

mlj

National standard criteria (e.g. Milliman, InterQual, etc)

mlj

Home­grown/customized criteria

mlj

None/case­by­case

**8. Please specify the national standard criteria used**

**9. To what proportion of elective inpatient procedures were the prior authorization criteria applied in 2011?**

mlj

All

mlj

Most

mlj

Some

mlj

None or Almost None

**10. What percent of elective inpatient procedures in 2011 were reviewed prior to admission**

**by someone with clinical expertise?**

**11. What percent of reviewed elective inpatient procedures were denied or modified in**

**2011?**

**12. What percent of patients received pre­admission education/planning for elective surgical procedures in 2011?**

**13. What was the number of concurrent review case manager FTEs dedicated to concurrent inpatient review in 2011?**

**14. What type of Length of Stay and Level of Care criteria did you use in 2011?**

mlj

National standard criteria (e.g. Milliman, InterQual, etc)

mlj

Home­grown/customized criteria

mlj

None/case­by­case

**15. Please specify the national standard criteria used**

**16. To what proportion of cases were the Length of Stay and Level of Care criteria applied**

**in 2011?**

mlj

All

mlj

Most

mlj

Some

mlj

None or Almost None

**17. How often did you receive a hospital census in 2011?**

mlj

Daily

mlj

Five to six times per week

mlj

Three to Four times per week

mlj

One to two times per week

mlj

Less than once a week

**18. What was the number of Medical Director FTEs dedicated to concurrent inpatient**

**review in 2011?**

**19. What was the frequency of RN case rounds per week in 2011?**

mlj

Daily

mlj

Five to six times per week

mlj

Three to Four times per week

mlj

One to two times per week

mlj

Less than once a week

**20. What was the frequency of on­site MD case rounds per week in 2011?**

mlj

Daily

mlj

Five to six times per week

mlj

Three to Four times per week

mlj

One to two times per week

mlj

Less than once a week

**21. What was the frequency of MD case rounds with RNs per week in 2011?**

mlj

Daily

mlj

Five to six times per week

mlj

Three to Four times per week

mlj

One to two times per week

mlj

Less than once a week

**22. What was the relationship with your MSO in 2011?**

mlj

MSO owns IPA/MG

mlj

IPA/MG owns MSO

mlj

MSO is independently owned and contracts with IPA/MG

mlj

Other (please specify)

**23. Did you have a direct contract with one or more hospitalist programs in 2011?**

mlj

Yes

mlj No

**24. Did you have a Scope of Practice agreement in place with one or more hospitalist**

**programs in 2011?**

mlj

Yes

mlj No

**25. How did you measure the effectiveness of the hospitalist program(s) in 2011?**

5

6

**26. Did the hospitalist program(s) have on­site coverage at night in 2011?**

mlj

Yes

mlj No

**27. Did the hospitalist program(s) have a post­discharge clinic in 2011 (i.e. follow­up**

**appointments directly with hospitalists following patients' discharge)?**

mlj

Yes

mlj No

**28. Did the contract and/or Scope of Practice include an agreement for hospitalists to**

**review and triage non­critical patients in the Emergency Department?**

mlj

Yes

mlj No

**29. Did the contract and/or Scope of Practice include an agreement on a standardized**

**hand­off procedure between hospitalist shifts?**

mlj

Yes

mlj No

**30. Please describe the standardized hand­off procedure between hospitalist shifts used**

**in 2011**

5

6

**31. Did any hospitals have an equity stake in the IPA/MG in 2011?**

mlj

Yes

mlj No

**32. Please specify which hospital(s) had an equity stake in the IPA/MG in 2011**

5

6

**33. Did you have an evidence­based disease management program in 2011?**

mlj

Yes

mlj No

**34. Was your disease management program opt in or opt out in 2011?**

mlj

Opt in

mlj

Opt out

**35. How many members were enrolled in the disease management program in 2011?**

**36. Please indicate for which chronic diseases the following disease management elements were in place in 2011**

|  | Diabetes | Asthma | Congestive heart failure | Depression |
| --- | --- | --- | --- | --- |
| Maintained an electronic list of patients with the illness | gfedc | gfedc | gfedc | gfedc |
| Provided the majority of physicians/practices with guideline­based reminders for services the patient should receive at the time of seeing the patient | fec | fec | fec | fec |
| Provided data to physicians/practices on the quality of care for patients | gfedc | gfedc | gfedc | gfedc |
| Sent reminders for preventive or follow­up care directly to majority of patients | fec | fec | fec | fec |
| Made specially trained health educators and/or nurses available to educate patients on how to manage their illness | gfedc | gfedc | gfedc | gfedc |
| Had nurse care managers on staff whose primary job was to coordinate and improve the quality of care for patients with illness | fec | fec | fec | fec |
| Other (please specify) |  |  |  |  |

5

6

**37. How did you measure the effectiveness of your disease management program in**

**2011?**

5

6

**38. Was your disease management program in 2011 administered by internal staff of your**

**IPA/MG or was it outsourced?**

mlj

Administered internally

mlj

Outsourced

**39. What was the number of RN FTEs dedicated to the disease management program in**

**2011?**

**40. Did you have an evidence­based case management program in 2011 (e.g. have nurses or other staff designated to call patients with reminders, have nurses or other staff designated to home visits, do care coordination, etc)?**

mlj

Yes

mlj No

**41. How many patients were enrolled in the evidence­based case management program in**

**2011?**

**42. What was the number of RN FTEs dedicated to case management in 2011?**

**43. Did you have an active ambulatory intensive care unit in 2011 for intensified primary care management of people with complex comorbidities?**

mlj

Yes

mlj No

**44. How many patients were in the active ambulatory intensive care program in 2011?**

**45. What other programs did you have in 2011 to improve care coordination for sick patients and/or complex cases?**

5

6

**46. In regards to your IPA/MG's active management of sick patients and/or complex cases**

**in 2011, what do you think worked well?**

5

6

**47. In regards to your IPA/MG's active management of sick patients and/or complex cases**

**in 2011, what do you think did not work well?**

5

6

**48. What was the number of IPA/MG FTEs designated for or dedicated to discharge**

**planning in 2011?**

**49. Did you have a practice in place in 2011 for discharge planning to begin at admission?**

mlj

Yes

mlj No

**50. Did you have a Scope of Practice agreement with hospitals in 2011 related to discharge**

**(e.g. (re)fill prescriptions, make follow­up appointment, etc)?**

mlj

Yes

mlj No

**51. Please describe what was in the discharge Scope of Practice agreement in 2011**

5

6

**52. What percent of patients were seen in an outpatient setting in 2011:**

within 7 days of discharge?

within 30 days of discharge?

**53. Did the hospitalist Scope of Practice include an agreement for hospitalists to be**

**involved in discharge planning in 2011?**

mlj

Yes

mlj No

**54. Who oversaw medication reconciliation upon discharge in 2011?**

mlj

Hospitalist

mlj

Pharmacist

mlj

Nurse

mlj

Other (please specify)

**55. Upon a patient’s discharge, did you have a process in 2011 of notifying patient’s PCP**

**or primary treating physician?**

mlj

Yes

mlj No

**56. Which of the following elements were in the notification to the patient’s PCP or primary**

**treating physician (check all that apply)?**

fec

Primary and secondary diagnoses

fec

Pertinent physical findings

fec

Results of procedures and laboratory tests

fec

New medication regimen and reasons for any changes

fec

Details of follow­up arrangements made

fec

Information given to the patient and family

fec

Test results pending at discharge

fec

Specific follow­up needs

fec

Other (please specify)

5

6

**57. Please describe criteria used to select/approve/hire providers to be a part of your**

**IPA/MG in 2011**

5

6

**58. How many physician employees/contracted providers were terminated in 2011?**

**59. How many times were physician employees/contracted providers terminated in 2011 for the following reasons:**

Concerns with quality of care

Concerns with excessive or costly care

Concerns with patient satisfaction

Formal disciplinary action/breach of contract

Conflicts over IPA policy

Malpractice claims

Inadequate demand for services from provider

**60. How many providers had exclusive contracts with your IPA/MG in 2011?**

5

6

**61. How many providers had a patient base of more than**

1000 members from your

IPA/MG in 2011?

400 members from your

IPA/MG in 2011?

200 members from your

IPA/MG in 2011?

**62. What percent of the IPA/MG capitation went to pay for PCP services in 2011?**

**63. Of the money going to PCP services in 2011, what percent was paid as fee­for­service?**

**64. Of the money going to PCP services in 2011, what percent was based on measures of physician productivity?**

**65. What percent of the IPA/MG capitation went to pay for specialist services in 2011?**

**66. Of the money going to specialist services in 2011, what percent was paid as fee­for­**

**service?**

**67. Of the money going to specialist services in 2011, what percent was based on measures of physician productivity?**

**68. Were providers given performance­based bonuses in 2011?**

mlj

Yes

mlj No

**69. Please indicate for both PCPs and specialists whether performance­based bonuses were calculated using any of the following criteria (check all that apply):**

PCPs Specialists

Hospital admittance, length of stay, or bed days/thousand rates

gfedc gfedc

ER visit rates fec fec Specialty referral gfedc gfedc Lab/imaging utilization fec fec Generics utilization rate gfedc gfedc

Preventive care utilization or quality

Chronic care quality measures

Patient experience measures

fec fec gfedc gfedc

fec fec

Other (please specify)

5

6

**70. How many seats did your board of directors have in 2011?**

**71. In 2011, how many board members were practicing PCPs?**

**72. In 2011, how many board members were practicing specialists?**

**73. In 2011, how many board members were hospital representatives (clinical or administrative)?**

**74. What was the average tenure of your board members as of 2011?**

**75. How many total patients (i.e. including non­BSC members) were in your IPA/MG in**

**2011?**

**76. How many total practices were in your IPA/MG in 2011?**

**77. How many total physicians were in your IPA/MG in 2011?**

**78. How many PCPs were in your IPA/MG in 2011?**

**79. In addition to Blue Shield, with which of the following insurance carriers did you contract in 2011 (check all that apply)?**

fec

Aetna

fec

Anthem Blue Cross

fec

CIGNA

fec

Health Net

fec

United Healthcare

fec

Medicare Advantage

fec

PPO (any)

**80. What was the percent of your physicians’ offices using an EHR in 2011?**

**81. Did your IPA/MG recommend a standard EHR system for all providers in 2011?**

mlj

Yes

mlj No

**82. Did your IPA/MG require a standard EHR system for all providers in 2011?**

mlj

Yes

mlj No

**83. Was there an IPA­ or MG­level health information exchange system in place to enable**

**doctors to communicate, send e­referrals, share records, etc, in 2011?**

mlj

Yes

mlj No

84.What percent of physicians' offices participated in the health information exchange system in 2011?
