# Supplementary material for: Physician organization care management capabilities associated with effective inpatient utilization management: a fuzzy set qualitative comparative analysis
Source: BMC Health Serv Res. 2014 Dec 3;14:582. doi: 10.1186/s12913-014-0582-5 (PMC4263202; doi:10.1186/s12913-014-0582-5)
Supplement: Additional file 3: — Data_Sheehy_Thygeson. [file 12913_2014_582_MOESM3_ESM.docx]

| name | bdk | alosmed | alossurg | medadmit | surgadmit | medreadmit | surgreadmit |
| --- | --- | --- | --- | --- | --- | --- | --- |
| 1 | 0.13 | 0.06 | 0.16 | 0.68 | 0.45 | 0.85 | 0.15 |
| 2 | 0.56 | 0.68 | 0.08 | 0.51 | 0.65 | 0.00 | 0.81 |
| 3 | 0.89 | 0.17 | 0.14 | 0.85 | 0.93 | 0.16 | 0.38 |
| 4 | 0.85 | 0.20 | 0.93 | 0.82 | 0.44 | 0.74 | 1.00 |
| 5 | 0.39 | 0.42 | 0.15 | 0.96 | 0.04 | 0.83 | 0.08 |
| 6 | 0.10 | 0.19 | 0.05 | 0.58 | 0.22 | 0.46 | 0.42 |
| 7 | 0.46 | 0.16 | 0.29 | 0.60 | 0.78 | 0.19 | 0.99 |
| 8 | 0.31 | 0.15 | 0.71 | 0.79 | 0.15 | 0.59 | 0.14 |
| 9 | 0.95 | 0.95 | 0.86 | 0.34 | 0.35 | 0.59 | 0.57 |
| 10 | 0.65 | 0.95 | 0.86 | 0.21 | 0.05 | 0.99 | 1.00 |
| 11 | 0.99 | 0.35 | 0.68 | 0.85 | 0.84 | 0.53 | 0.67 |
| 12 | 0.98 | 0.86 | 0.96 | 0.65 | 0.30 | 0.79 | 0.64 |
| 13 | 0.78 | 0.85 | 0.16 | 0.06 | 0.87 | 0.25 | 0.36 |
| 14 | 0.36 | 0.23 | 0.95 | 0.50 | 0.13 | 0.62 | 0.99 |

| rawdxcg | hospnum | concon | rnrdfreq | concrn | hosp | hospeff |
| --- | --- | --- | --- | --- | --- | --- |
| 0.31 | 1.00 | 0.67 | 0.33 | 0.33 | 0.00 | 0.67 |
| 0.20 | 0.80 | 0.00 | 0.67 | 0.00 | 0.33 | 0.00 |
| 0.06 | 1.00 | 0.67 | 1.00 | 0.67 | 0.33 | 0.67 |
| 0.84 | 0.20 | 1.00 | 0.67 | 0.67 | 1.00 | 0.67 |
| 0.37 | 0.60 | 0.00 | 0.67 | 0.00 | 0.33 | 0.67 |
| 0.85 | 0.40 | 1.00 | 0.67 | 0.67 | 1.00 | 0.67 |
| 0.90 | 0.40 | 0.00 | 0.67 | 0.00 | 0.33 | 0.67 |
| 0.68 | 0.40 | 0.67 | 0.67 | 0.67 | 0.33 | 0.00 |
| 0.18 | 0.20 | 1.00 | 0.67 | 0.67 | 1.00 | 0.67 |
| 0.57 | 0.40 | 1.00 | 0.67 | 0.67 | 1.00 | 1.00 |
| 0.36 | 0.80 | 0.67 | 0.67 | 0.67 | 1.00 | 1.00 |
| 0.62 | 1.00 | 0.33 | 1.00 | 0.33 | 1.00 | 0.67 |
| 0.37 | 0.20 | 1.00 | 1.00 | 1.00 | 1.00 | 1.00 |
| 0.49 | 1.00 | 0.00 | 0.00 | 0.00 | 1.00 | 0.33 |

| hospandeff | discharge | disproc | disand | prior | disnot |
| --- | --- | --- | --- | --- | --- |
| 0.00 | 0.67 | 0.67 | 0.67 | 1.00 | 0.33 |
| 0.00 | 0.33 | 0.67 | 0.33 | 1.00 | 0.00 |
| 0.33 | 0.67 | 0.67 | 0.67 | 1.00 | 1.00 |
| 0.67 | 0.67 | 1.00 | 0.67 | 0.67 | 1.00 |
| 0.33 | 0.00 | 0.67 | 0.00 | 0.67 | 0.00 |
| 0.67 | 0.33 | 0.00 | 0.00 | 0.00 | 0.67 |
| 0.33 | 0.33 | 0.00 | 0.00 | 1.00 | 0.00 |
| 0.00 | 0.67 | 0.67 | 0.67 | 1.00 | 0.00 |
| 0.67 | 1.00 | 1.00 | 1.00 | 1.00 | 0.33 |
| 1.00 | 0.67 | 1.00 | 0.67 | 0.67 | 0.33 |
| 1.00 | 0.67 | 1.00 | 0.67 | 1.00 | 0.33 |
| 0.67 | 1.00 | 0.67 | 0.67 | 0.67 | 0.33 |
| 1.00 | 0.67 | 1.00 | 0.67 | 1.00 | 0.33 |
| 0.33 | 0.00 | 1.00 | 0.00 | 0.33 | 0.33 |

| hospnite | hosped | hospniteored | dismgmt | casefte | ucctot | uccnonpeak | mdrdfreq |
| --- | --- | --- | --- | --- | --- | --- | --- |
| 1.00 | 0.00 | 1.00 | 0.67 | 0.86 | 0.00 | 0.00 | 0.67 |
| 1.00 | 0.00 | 1.00 | 0.00 | 0.00 | 0.34 | 0.18 | 0 |
| 1.00 | . | 1.00 | 0.67 | 0.17 | 0.43 | 0.22 | 0 |
| 1.00 | 1.00 | 1.00 | 1.00 | 1.00 | 0.22 | 0.22 | 1 |
| 1.00 | 0.00 | 1.00 | 0.67 | 0.99 | 0.58 | 0.34 | 0 |
| 1.00 | 1.00 | 1.00 | 1.00 | 0.57 | 0.69 | 0.60 | 1 |
| 1.00 | 1.00 | 1.00 | 0.67 | 0.33 | 0.99 | 1.00 | 0 |
| 1.00 | 0.00 | 1.00 | 0.33 | 0.00 | 0.00 | 0.00 | 0 |
| 0.00 | 1.00 | 1.00 | 0.67 | 0.65 | 0.23 | 0.47 | 0.33 |
| 1.00 | 1.00 | 1.00 | 1.00 | 1.00 | 0.57 | 0.44 | 1 |
| 1.00 | 1.00 | 1.00 | 0.00 | 0.97 | 0.35 | 0.19 | 0 |
| 1.00 | 1.00 | 1.00 | 0.33 | 0.02 | 0.79 | 0.78 | 0 |
| 0.00 | 0.00 | 0.00 | 0.67 | 0.65 | 0.00 | 0.00 | 0 |
| 1.00 | 1.00 | 1.00 | 0.33 | 0.00 | 0.61 | 0.37 | 0 |
