# Supplementary material for: Physician organization care management capabilities associated with effective inpatient utilization management: a fuzzy set qualitative comparative analysis
Source: BMC Health Serv Res. 2014 Dec 3;14:582. doi: 10.1186/s12913-014-0582-5 (PMC4263202; doi:10.1186/s12913-014-0582-5)
Supplement: Additional file 4: — Data dictionary_Sheehy_Thygeson. [file 12913_2014_582_MOESM4_ESM.docx]

|  | |  | |
| --- | --- | --- | --- |
| Condition Description | Condition Name | | Condition Calibration Function |
| Bed Days per Thousand Members | bdk | | Continuous (indirect)—a fuzzy score of 1 indicates well-managed bed days per thousand |
| Average Length of Stay, medical | alosmed | | Continuous (indirect)—a fuzzy score of 1 indicates low average length of stay |
| Average Length of Stay, surgical | alossurg | | Continuous (indirect)—a fuzzy score of 1 indicates low average length of stay |
| Medical Admissions per Thousand Members | medadmit | | Continuous (indirect)—a fuzzy score of 1 indicates a low number of admissions |
| Medical Readmissions per Thousand Members | medreadmit | | Continuous (indirect)—a fuzzy score of 1 indicates a low number of admissions |
| Surgical Admissions per Thousand Members | surgadmit | | Continuous (indirect)—a fuzzy score of 1 indicates a low number of admissions |
| Surgical Readmissions per Thousand Members | surgreadmit | | Continuous (indirect)—a fuzzy score of 1 indicates a low number of readmissions |
| Illness burden (DxCG score) | rawdxcg | | Continuous (direct)—a fuzzy score of 1 indicates a high illness burden (high DxCG score) |
| Average number of total urgent care center open hours | ucctot | | Continuous (indirect)—a fuzzy score of 1 indicates a high number of open hours per in-network UCC facility |
| Number of in-area hospitals | hospnum | | 0 = 1 hospital;  0.33 = 2 hospitals;  0.67 = 3 hospitals;  1 = 4 or more hospitals; |
| Concurrent review process by PO RNs | concrn | | Intersection (minimum) of on-site and frequent review |
| RN on-site concurrent review method | concon | | 1: All in-area RN concurrent review is conducted on-site;  0.67: High-volume hospitals have on-site concurrent review, other facilities are telephonic review;  0.33: Some concurrent review is on-site;  0: All concurrent review is telephonic/electronic OR no direct PO role in active concurrent review |
| RN rounds frequency (on-site or telephonic/electronic) | rnrdfreq | | 1: RN rounds occur 7 days per week;  0.67: RN rounds occur 5 days per week;  0.33: RN rounds occur 3-4 days per week;  0: RN rounds occur 1-2 days per week |
| Terms of Hospitalist-PO relationship | hospandeff | | Intersection (minimum) of relationship with hospitalist program and evaluation of hospitalist effectiveness |
| Strength of relationship with hospitalist program(s) | hosp | | 1: PO has both a contract and Scope of Practice with one or more hospitalist programs OR has a contract that functions as a Scope of Practice (specifying responsibilities for utilization management related behaviors);  0.33: PO has a contract but no Scope of Practice with one or more hospitalist programs;  0: PO has no contract or Scope of Practice with any hospitalist programs |
| Evaluation of hospitalist effectiveness | hospeff | | 1: PO has regular, robust evaluation of UM metrics AND/OR UM data regularly shared with hospitalist representative(s);  0.67: All UM data regularly reviewed internally;  0.33: UM data reviewed sometimes;  0: No criteria or method in place to evaluate hospitalist effectiveness |
| Hospitalist after-hour or ED coverage | hospniteored | | Union (maximum) of hospitalist on-site coverage at night and triage/review of non-critical patients in the Emergency Department |
| Hospitalist program has on-site coverage at night | hospnite | | 1: Yes;  0: No |
| Hospitalist program triages/reviews non-critical patients in Emergency Department | hosped | | 1: Yes;  0: No |
| PO role in discharge planning | disand | | Intersection (minimum) of guidelines for discharge planning process and PO staff involvement in discharge planning process |
| Guidelines for discharge planning process | discharge | | 1: Scope of Practice with hospitals/hospitalists related to discharge AND practice in place for discharge to begin at admission;  0.67: Either Scope of Practice or practice for discharge to begin at admission (but not both);  0: Neither a Scope of Practice with hospitals nor practice in place for discharge to begin at admission |
| PO staff involvement in discharge planning process | disproc | | 1: PO oversees discharge full process for own patients;  0.67: PO staff coordinates with hospital staff and plays lead role (e.g. close contact with individual patients, plays primary role in transition to outpatient setting, etc);  0.33: PO staff coordinates with hospital staff and plays backup role (e.g. most contact with hospital is telephonic, IPA defers to hospital on issues of discharge planning);  0: No PO role in discharge planning (e.g. no contact with patient until after they leave hospital, no telephonic coordination with hospital staff regarding details of discharge of patients, etc) |
| Prior authorization procedure | prior | | 1: National criteria always applied, always reviewed by someone with clinical experience;  0.67: National criteria mostly applied, all/most reviewed by someone with clinical experience;  0.33: National criteria mostly applied, sometimes reviewed by someone with clinical experience OR National criteria sometimes applied, mostly reviewed by someone with clinical experience;  0: National standard sometimes applied, sometimes/rarely by someone with clinical experience |
| Discharge notification sent to patients’ primary care physician | disnot | | 1: PO sends notification directly to PCPs and it includes all/nearly all information;  0.67: PO sends notification directly to PCPs but it includes only basic clinical information;  0.33: PO plays secondary role OR has "passive" notification system in place (e.g. clinical hub connects hospital and PO doctors);  0: No PO involvement |
| Disease management program | dismgmt | | 1:PO has disease management program and methodology that is distinct from general case management approach;  0.67: PO actively manages chronic patients as a part of broader case management program;  0.33: PO "passively" manages chronic patients (e.g. makes resources available but does not actively manage/coordinate care);  0: PO has no program OR refers all patients to health plans |
| PO FTEs dedicated to case management | casefte | | Continuous (indirect)—a fuzzy score of 1 indicates a low ratio of FTEs to number of members enrolled in case management |
| Average number of non-peak (i.e. non M-F 8:00-5:00) urgent care center open hours | uccnonpeak | | Continuous (indirect)—a fuzzy score of 1 indicates a high number of non-peak open hours per in-network UCC facility |
| Frequency of PO Medical Director on-site rounds | mdrdfreq | | 1: 5 or more times per week;  0.67: 3-4 times per week;  0.33: 1-2 times per week;  0: Fewer than once per week on average |
