# Supplementary material for: Physician organization care management capabilities associated with effective inpatient utilization management: a fuzzy set qualitative comparative analysis
Source: BMC Health Serv Res. 2014 Dec 3;14:582. doi: 10.1186/s12913-014-0582-5 (PMC4263202; doi:10.1186/s12913-014-0582-5)
Supplement: Additional file 5: — Figure S1_Sheehy_Thygeson. [file 12913_2014_582_MOESM5_ESM.docx]

**Figure S1. Logic Model**

Average Length of Stay

Medical Admissions

Surgical Days/Thousand

Medical Days/Thousand

Total Bed Days/Thousand

Disease Management Case Management Urgent Care Availability Medical Readmissions

Concurrent Review Discharge Planning Hospitalist Contracting

Prior Authorization
Surgical Readmissions

Surgical Admissions

Health Risk (DxCG)
