# Supplementary material for: Physician organization care management capabilities associated with effective inpatient utilization management: a fuzzy set qualitative comparative analysis
Source: BMC Health Serv Res. 2014 Dec 3;14:582. doi: 10.1186/s12913-014-0582-5 (PMC4263202; doi:10.1186/s12913-014-0582-5)
Supplement: Additional file 6: — Online Appendix_Sheehy_Thygeson. [file 12913_2014_582_MOESM6_ESM.docx]

**Online Appendix**

Table A1

|  | Minimum | Maximum | Average |
| --- | --- | --- | --- |
| Bed-Days per Thousand | 124.36 | 199.42 | 153.99 |
| Medical Average Length of Stay | 2.27 | 5.39 | 3.93 |
| Surgical Average Length of Stay | 3.30 | 5.21 | 4.18 |
| Medical Admissions per Thousand | 13.96 | 26.58 | 20.33 |
| Surgical Admissions per Thousand | 12.16 | 23.56 | 18.23 |

To conduct the Fisher’s exact tests we created six dichotomous variables: low BDk, well-managed BDk, and low medical and surgical ALOS and APk. POs with a BDk fuzzy score of 0.5 or more were assigned a “low BDk” value of 1, with all other POs coded as 0. For each PO we also calculated fuzzy scores for each of the three solution paths to achieve low BDk (see Table 2, Model 1). POs with a fuzzy score of 0.5 or greater on at least one of the solution paths was assigned a “well-managed BDk” score of 1, and all other POs were coded as 0. Finally, for medical and surgical ALOS and APk, POs were assigned a value of 1 for fuzzy scores greater than or equal to 0.5 (e.g. fuzzy membership in “low medical ALOS” > 0.5) and values of 0 otherwise. Using these variables we conducted Fisher’s exact tests to test the hypothesis of group membership in low BDk based on each of four criteria: well-managed BDk, low medical ALOS or APk, and low surgical ALOS and APk (Tables A2–A6). Only two tests are statistically significant: all groups achieving at least one of the BDk solution paths as well as those with low medical ALOS also had low BDk.

The well-managed BDk and low medical and surgical ALOS and APk dichotomous variables were also used to differentiate group for the test of the difference in mean BDk (see Table 4).

| Table A2–A6: Fischer Exact Tests | |
| --- | --- |
| \| Table A2: BDk Solution Paths \| \| \| \| \| --- \| --- \| --- \| --- \| \| Low Bed Days per thousand \| Well-Managed (any solution path) \| \| \| \| No \| Yes \|  \| \| No \| 6 \| 0 \| 6 \| \| Yes \| 2 \| 6 \| 8 \| \|  \| 8 \| 6 \| 14 \| \| Fisher’s exact = 0.010  1-sided Fisher’s exact = 0.009 \| \| \| \| | \| Table A3: Medical Length of Stay \| \| \| \| \| --- \| --- \| --- \| --- \| \| Low Bed Days per thousand \| Low medical ALOS \| \| \| \| No \| Yes \| Total \| \| No \| 6 \| 0 \| 6 \| \| Yes \| 3 \| 5 \| 8 \| \|  \| 9 \| 5 \| 14 \| \| Fisher’s exact 0.031  1-sided Fisher’s exact = 0.028 \| \| \| \| |
| \| Table A4: Surgical Admits \| \| \| \| \| --- \| --- \| --- \| --- \| \| Low Bed Days per thousand \| Low surgical APk \| \| \| \| No \| Yes \| Total \| \| No \| 5 \| 1 \| 6 \| \| Yes \| 4 \| 4 \| 8 \| \|  \| 9 \| 5 \| 14 \| \| Fisher’s exact = 0.301  1-sided Fisher’s exact = 0.238 \| \| \| \| | \| Table A5: Surgical Length of Stay \| \| \| \| \| --- \| --- \| --- \| --- \| \| Low Bed Days per thousand \| Low surgical ALOS \| \| \| \| No \| Yes \| Total \| \| No \| 4 \| 2 \| 6 \| \| Yes \| 3 \| 5 \| 8 \| \|  \| 7 \| 7 \| 14 \| \| Fisher’s exact = 0.592  1-sided Fisher’s exact = 0.296 \| \| \| \| |
| \| \| Table A6: Medical Admits \| \| \| \| \| --- \| --- \| --- \| --- \| \| Low Bed Days per thousand \| Low medical APk \| \| \| \| No \| Yes \| Total \| \| No \| 0 \| 6 \| 6 \| \| Yes \| 3 \| 5 \| 8 \| \|  \| 3 \| 11 \| 14 \| \| Fisher’s exact = 0.209  1-sided Fisher’s exact = 0.154 \| \| \| \| \| \| --- \| --- \| --- \| --- \| --- \| --- \| --- \| --- \| --- \| --- \| --- \| --- \| --- \| --- \| --- \| --- \| --- \| --- \| --- \| --- \| --- \| --- \| --- \| --- \| --- \| --- \| --- \| --- \| | |
